# Tailored flavoproteins acting as light-driven spin machines pump nuclear hyperpolarization

**DOI:** 10.1038/s41598-020-75627-z

**Published:** 2020-10-29

**Authors:** Yonghong Ding, Alexey S. Kiryutin, Ziyue Zhao, Qian-Zhao Xu, Kai-Hong Zhao, Patrick Kurle, Saskia Bannister, Tilman Kottke, Renad Z. Sagdeev, Konstantin L. Ivanov, Alexandra V. Yurkovskaya, Jörg Matysik

**Affiliations:** 1 Institut für Analytische Chemie, Universität Leipzig, Linnéstr. 3, 04103 Leipzig, Germany; 2 International Tomography Center, Siberian Branch of Russian Academy of Sciences, Institutskaya, 3a, Novosibirsk, 630090 Russia; 3 Novosibirsk State University, Pirogova 1, Novosibirsk, 630090 Russia; 4 State Key Laboratory of Agricultural Microbiology, Huazhong Agricultural University, Wuhan, 430070 China; 5 Physikalische und Biophysikalische Chemie, Universität Bielefeld, Universitätsstr. 25, 33615 Bielefeld, Germany

**Keywords:** Biochemistry, Biophysics, Chemical biology, Chemistry

## Abstract

The solid-state photo-chemically induced dynamic nuclear polarization (photo-CIDNP) effect generates non-Boltzmann nuclear spin magnetization, referred to as hyperpolarization, allowing for high gain of sensitivity in nuclear magnetic resonance (NMR). Well known to occur in photosynthetic reaction centers, the effect was also observed in a light-oxygen-voltage (LOV) domain of the blue-light receptor phototropin, in which the functional cysteine was removed to prevent photo-chemical reactions with the cofactor, a flavin mononucleotide (FMN). Upon illumination, the FMN abstracts an electron from a tryptophan to form a transient spin-correlated radical pair (SCRP) generating the photo-CIDNP effect. Here, we report on designed molecular spin-machines producing nuclear hyperpolarization upon illumination: a LOV domain of aureochrome1a from *Phaeodactylum tricornutum*, and a LOV domain named 4511 from *Methylobacterium radiotolerans* (*Mr*4511) which lacks an otherwise conserved tryptophan in its wild-type form. Insertion of the tryptophan at canonical and novel positions in *Mr*4511 yields photo-CIDNP effects observed by ^15^N and ^1^H liquid-state high-resolution NMR with a characteristic magnetic-field dependence indicating an involvement of anisotropic magnetic interactions and a slow-motion regime in the transient paramagnetic state. The heuristic biomimetic design opens new categories of experiments to analyze and apply the photo-CIDNP effect.

## Introduction

The solid-state photo-CIDNP (photo-chemically induced dynamic nuclear polarization) effect^1–3^ allows to enhance nuclear magnetic resonance (NMR) signals by a build-up of transient nuclear spin-hyperpolarization caused by light-induced transient spin-correlated radical pairs (SCRPs) in electron-transfer proteins^4,5^. The effect was discovered by Zysmilich and McDermott in 1994 by performing ^15^N magic-angle spinning (MAS) NMR studies on an isolated, frozen and quinone-blocked photosynthetic reaction center protein of the purple bacterium *Rhodobacter sphaeroides*^6^. Since its discovery, the effect has been observed in plenty of other photosynthetic reaction centers (RCs) of plants^7–10^, algae^11,12^, diatoms^13,14^, purple bacteria^15–18^, heliobacteria^19,20^, and green sulfur bacteria^21^. The spin-chemical machinery pumping nuclear polarization has been probed by field-dependent^22,23^, time-resolved^24–26^, and preparation-dependent^27,28^ experiments, and is interpreted by the occurrence of up to three mechanisms running in parallel^22,24,29^: In the Differential Relaxation (DR) mechanism, also called “cyclic reactions mechanism”, the symmetry between both decay branches is broken due to a difference in relaxation caused by the paramagnetic molecular triplet state^30–32^. In the electron–electron nuclear Three-Spin Mixing (TSM) mechanism, symmetry breaking is driven by the pseudosecular part of the hyperfine interaction during the evolution of the SCRP^33,34^. The differences in decay kinetics between the SCRP in its singlet and its triplet state cause the Differential Decay (DD) mechanism^35^. Recently, the coherent electron–electron–nuclear spin-dynamics has been described in terms of a unified theoretical approach, based on level crossings (LCs) and level anti-crossings (LACs)^36–38^, which allowed the establishment of CIDNP sign rules and to determine conditions optimal for polarization formation.

Besides photosynthetic RCs, there is another class of proteins acting as molecular spin-machines pumping non-Boltzmann nuclear spin-state upon illumination: biological photoreceptors that contain a flavin cofactor as chromophore with characteristic absorption maxima in the UV-A and blue light regions. Merely one family of flavoproteins, phototropins, was reported to show the photo-CIDNP effect^39–41^. Generally, phototropins are blue-light receptors that harbor two light-sensing light-oxygen-voltage (LOV) domains, each of which incorporates one flavin mononucleotide (FMN) cofactor non-covalently. Upon excitation, the FMN in a wild-type LOV domain of phototropin undergoes adduct formation with a close-by conserved cysteine^42,43^. However, so far, no photo-CIDNP effect was observed from a wild-type LOV domain. The first successful solid-state photo-CIDNP effect observation was demonstrated for a frozen cysteine-lacking LOV domain of phototropin from the green alga *Chlamydomonas reinhardtii* (*Cr*phot-LOV1-C57S)^41,44^. In this case, the photo-excited FMN undergoes inter-system crossing to the triplet state, causes intra-protein electron transfer from a tryptophan to the FMN and thus gives rise to formation of a transient SCRP. Spin evolution of this SCRP allows for the build-up of the solid-state photo-CIDNP selectively on FMN and tryptophan. In *Cr*phot-LOV1-C57S, the edge-to-edge distance ($r_{FW}$) between FMN (F) and tryptophan (W) is ~ 11 Å. Remarkably, Weber et al. first observed photo-CIDNP in a similar LOV protein, the LOV2 domain of phototropin 1 from oat, *Avena sativa* (*As*phot1-LOV2-C450A), by ^13^C liquid-state NMR^40,45,46^. The crystal structure of *As*phot1-LOV2-C450A is almost identical to that of *Cr*phot-LOV1-C57S, given that they share 47% of amino acid sequences. The $r_{FW}$ value is very similar for the two proteins. This is a very rare example that a nuclear-spin hyperpolarization effect generated in one system could be observed by both liquid-state and solid-state NMR. To explore the action of anisotropic mechanisms in the liquid sample that are expected to be sensitive to the magnetic field strength^44,46^, a solution-state NMR spectrometer equipped with a field-cycling device^47^ was employed to study ^1^H, ^13^C and ^15^N photo-CIDNP generated in *Cr*phot-LOV1-C57S within a broad field range from 0.01–9.4 T^39^.

The LOV domain is the blue-light mediating motif of not only phototropin but also of some other proteins, e.g., aureochromes^48^. Aureochromes represent a class of LOV proteins with an unusual inversed domain arrangement, as they carry, in contrast to the majority of LOV-domain proteins, the functional/signaling domain N-terminally to the LOV domain and not C-terminally; they exhibit great potential in controlling DNA binding^49^ as a natural optogenetic module. Despite a different domain organization of the full-length protein, the LOV domain of aureochromes seem to show a high degree of conservation to canonical LOV domains with respect to sequence and secondary structure^50^. Like in other LOV domains, the optical excitation of FMN chromophore leads to formation of an adduct with a conserved cysteine residue. Furthermore, a recently discovered flavoprotein from the radiation-resistant bacterium *Methylobacterium radiotolerans*, *Mr*4511, unusually lacks the single tryptophan conserved in 75% of LOV domains^51^. Due to the absence of tryptophan to quench the triplet state of FMN, ^3^FMN, after excitation by light, the cysteine-lacking mutant of *Mr*4511 can serve as an efficient singlet-oxygen generator^51^. The variety of LOV domains raises the question whether LOV domains can be generally constructed in such a way that they show a photo-CIDNP effect.

The aim of this work was to extend the range of biological systems amenable to the solid-state photo-CIDNP effect. To reach this goal, we at first produced a cysteine-lacking LOV domain of aureochrome1a from the diatom *Phaeodactylum tricornutum* (*Pt*aureo1a-LOV-C287S)^52,53^ and show that it generated ^1^H-, ^13^C-, and ^15^N photo-CIDNP effects in aqueous solution. By using a field-cycling system, the magnetic field-dependencies of the ^1^H, ^13^C, and ^15^N hyperpolarization effects have been obtained revealing that the magnetic field for maximal photo-CIDNP depends on the nuclear gyromagnetic ratio. Such a behavior is a characteristic feature of the solid-state photo-CIDNP effect^36,39^, closely resembling that of *Cr*phot-LOV1-C57S. Based on this result, we propose that, like in phototropin, the anisotropic magnetic interactions might play a decisive role in photo-CIDNP formation in the LOV domain of aureochrome in solution.

Furthermore, in the heuristic approach, biomimetic protein design is used to control the conditions for the occurrence of the photo-CIDNP effect. We employed *Mr*4511 for an extended protein mutation strategy allowing us to change parameters such as the distance between the partners governing the electron transfer reactions that give rise to SCRP formation and recombination, and to tune magnetic parameters of the SCRP. By doing so, we were able to probe the spin dynamics in the SCRP by magnetic-field dependent photo-CIDNP studies. We could also elucidate the role of different photo-CIDNP mechanisms that are responsible for the formation of nuclear spin-polarization. Last but not least, we discovered a non-tryptophan-induced photo-CIDNP effect generated by the cysteine-devoid *Mr*4511, in which tryptophan is absent.

## Results

To rationalize the key properties of molecular spin-machines that can be used to generate photo-CIDNP, we proposed a design strategy based on mutations, supported by field-dependent CIDNP studies. By using various mutations, as described below, we were able to vary the distance between the electron donor and acceptor. In this way, we affected the rate of SCRP formation and recombination, and also varied the electron–electron spin–spin interaction in the SCRP. To probe the reaction and spin dynamics in the SCRP, we used the field dependence of photo-CIDNP.

### Screening LOV domains for induction of photo-CIDNP

Aiming for designed molecular spin-machines producing light-induced nuclear hyperpolarization, we have designed a series of protein mutants, which will be presented in parts (i)—(iii) (Table 1).

**Table 1:** LOV proteins employed for photo-CIDNP NMR in this work.

Mutants	$r_{FW}$ (Å)^a^	Species	Amino acid numbers	Weight (kDa)	Structure	Percentage identity^b^
*Cr*phot-LOV1-C57S	~ 11	*C. reinhardtii*	16–133	~ 15	PDB 1N9L	–
*Pt*aureo1a-LOV-C287S	~ 11	*P. tricornutum*	238–378	~ 18	PDB 5A8B	53%
*Mr*4511-C71S-F130W	~ 6	*M. radiotolerans*	1–164	~ 18	This work^a^ (Fig. 1)	43%
*Mr*4511-C71S-Y116W	~ 9					
*Mr*4511-C71S-Q112W	~ 11					
*Mr*4511-C71S-Y129W	~ 11					
*Mr*4511-C71S-K57W	~ 17					
*Mr*4511-C71S	–					

^a^The FMN-Trp distance ($r_{FW}$) is given as edge-to-edge distance and estimated based on a structural model obtained by SWISS-MODEL^55^.

^b^The percentage identity is compared with the amino-acid sequence of *Cr*phot-LOV1-C57S.

**Figure 1:**
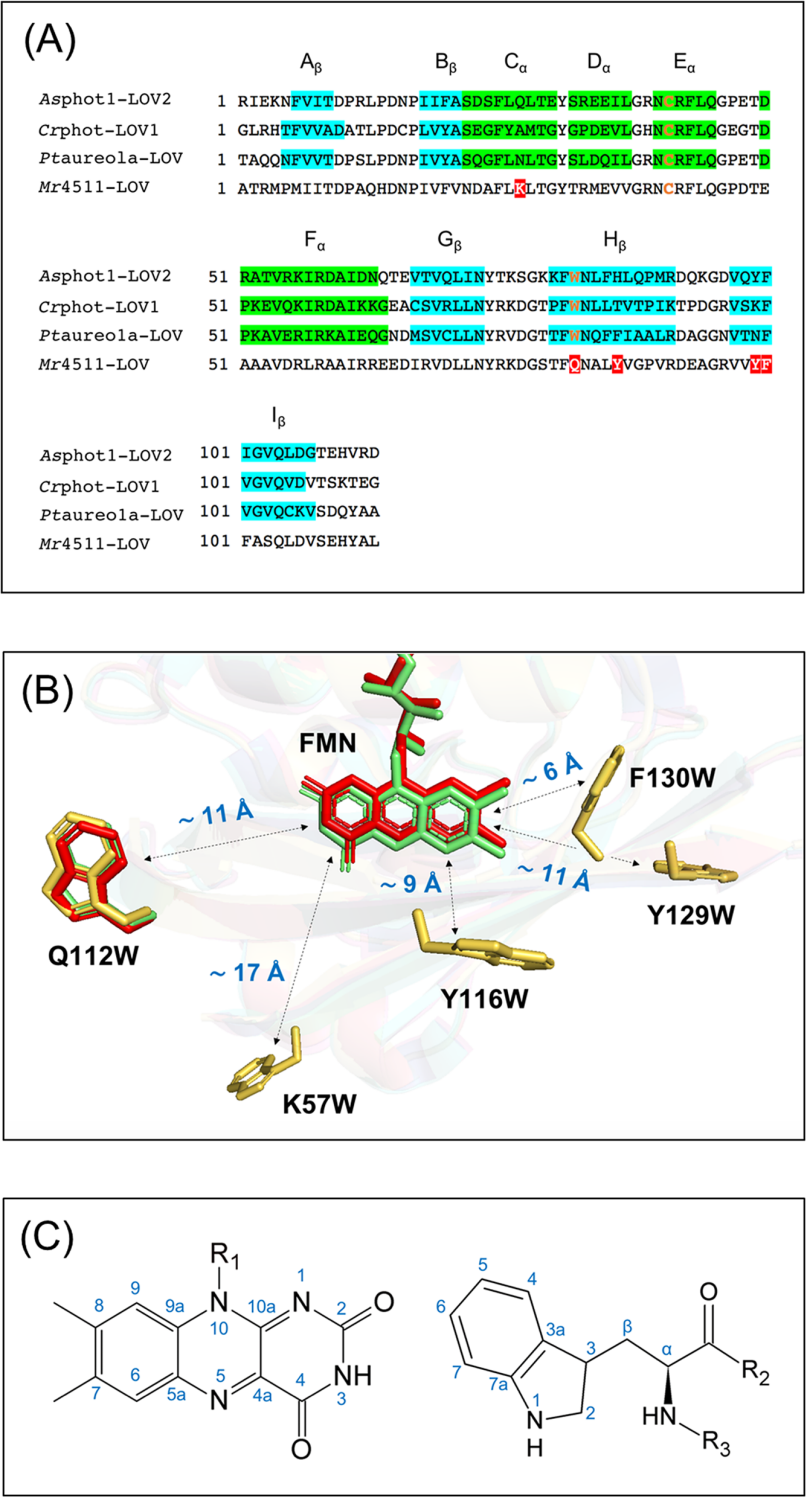
(**A**) Alignments of the amino-acid sequence of wild-type phototropin 1-LOV2 from *Avena sativa* (*As*phot1-LOV2), phototropin-LOV1 from *Chlamydomonas reinhardtii* (*Cr*phot-LOV1), aureochrome1a-LOV from *Phaeodactylum tricornutum* (*Pt*aureo1a-LOV), and 4511 from *Methylobacterium radiotolerans* (*Mr*4511): segments highlighted in green and blue refer to α-helices, and β-sheets, respectively, indicating the secondary structure of the proteins. The conserved positions of cysteine located in E_α_ and tryptophan in H_β_ in the LOV domains are typeset in orange. The five positions to introduce tryptophan in *Mr*4511 via mutagenesis are highlighted in red. (**B**) Alignment of the crystal structures of *Cr*phot-LOV1 (PDB: 1N9L, green)^56^, *Pt*aureo1a-LOV (PDB: 5A8B, red)^57^, and the simulated structure of *Mr*4511 (yellow) without FMN. The simulation is performed with SWISS-MODEL based on the crystal structure of aureochrome1a-LOV from *Vaucheria frigida* (PDB entry: 3UE6)^58^. The information about structural modeling is listed in Supplementary Information Table S1. Five mutants of cysteine-lacking *Mr*4511 were generated, one with tryptophan placed at the canonical position, Q112W, $r_{FW}$ ~ 11 Å, the other four at non-canonical positions F130W, Y116W, Y129W and K57W with increasing $r_{FW}$. The figure was created by the PyMOL Molecular Graphics System, Version 1.2r3pre, Schrödinger, LLC. (**C**) IUPAC numbering of atomic positions in the isoalloxazine ring of FMN and the side chain of tryptophan.
(ii) Formation of an SCRP by electron transfer to excited FMN can occur if a nearby tryptophan is present to act as the electron donor. It has been shown that the amino acid tryptophan is able to provide this function^41,54^. Therefore, in the LOV protein *Mr*4511, lacking the conserved tryptophan, we introduced a tryptophan at its canonical position Q112 by mutation resulting in the *Mr*4511-C71S-Q112W double mutant. Previously, transient absorption experiments have been used to test the function of these mutants. In *Mr*4511, when the cysteine residue was mutated to serine (C71S) or glycine (C71Q) and no tryptophan was present, the lifetime of ^3^FMN, $\tau_{T}$, was around 240 μs^51^. Introduction of tryptophan to the canonical position, *Mr*4511-C71S-Q112W, gave rise to faster quenching of ^3^FMN reducing $\tau_{T}$ to ~ 24 μs^51^, a value very close to ~ 27 μs observed in *Cr*phot-LOV1-C57S ($r_{FW}$ ~ 11 Å)^43^. Hence, the double mutant *Mr*4511-C71S-Q112W ($r_{FW}$ ~ 11 Å) is the second candidate for the generation of the solid-state photo-CIDNP effect.
(iii) Finally, we introduced the electron-donating tryptophan at non-canonical positions. The introduction of tryptophan to a new position of the protein allows to change the distance between FMN and tryptophan, their relative orientation and chemical environments and, therefore, to affect the kinetic and magnetic parameters, critical for the formation of the solid-state photo-CIDNP effect. It is difficult to fine-tune all relevant reaction and magnetic resonance parameters simultaneously, therefore, we focused on creating mutants with different $r_{FW}$. Lacking a crystal structure of *Mr*4511, the design relied on a structural model created using SWISS-MODEL and the crystal structure of aureochrome1a-LOV (PDB: 3UE6) from a eukaryotic photosynthetic stramenopile as template^58^. The report of the modeling parameters is provided in Supplementary Information Table S1. Additionally, comparison of the amino-acid sequence (Fig. 1A) allowed us to predict the occurrence of α-helix and β-sheet secondary structures and to reconstruct the tertiary structure of *Mr*4511. As targets for mutation, we considered amino acids that do not interact with the FMN^50^ and also have a bulky side chain similar as tryptophan. Using these ideas, we have designed the following mutants with different positions of tryptophan with various $r_{FW}$ values: *Mr*4511-C71S-F130W (~ 6 Å), *Mr*4511-C71S-Y116W (~ 9 Å), *Mr*4511-C71S-Y129W (~ 11 Å) and *Mr*4511-C71S-K57W (~ 17 Å) (Table 1).

(i) So far, the occurrence of the solid-state photo-CIDNP effect was limited to cysteine-lacking LOV domains of phototropin^39–41^; for this reason, here we explored other potential LOV-based light-induced hyperpolarization generators. Alignment and comparison of the amino-acid sequences of *As*phot1-LOV2, *Cr*phot-LOV1 and *Pt*aureo1a-LOV show about 50% of identity (Fig. 1A) and the crystal structures show almost identical tertiary structures (Fig. 1B). In particular, the distance between FMN and tryptophan (Fig. 1C), which determines the strength of the spin–spin coupling in the SCRP and is therefore central to generate photo-CIDNP, is nearly the same, being approximately 11 Å. Therefore, we used the mutant *Pt*aureo1a-LOV-C287S ($r_{FW}$ ~ 11 Å) for the liquid state photo-CIDNP NMR experiment, in which the conserved cysteine 287 is replaced by serine.

Another aspect relevant for rational design of a biomimetic light-driven spin-machine for production of photo-CIDNP is the possibility to introduce isotope labels. In particular, for the measurement of the ^15^N photo-CIDNP we employed ^15^NH_4_Cl as the sole nitrogen source in the bacterial growth medium during protein expression and produced uniformly ^15^N-labelled protein and cofactor (see “Methods”). For the ^13^C photo-CIDNP experiment, different labelling strategies were previously applied, either by incorporating the ^13^C-labelled FMN into a natural abundant protein moiety of the phototropin-LOV domain^40,46^ or by selective ^13^C-labelling of the single tryptophan of the phototropin-LOV domain^39^. This enables unambiguous assignment of hyperpolarized ^13^C signals and analysis of the photo-CIDNP effect generated by electron donor and acceptor separately. A complete picture of the effect, however, involving both electron donor and acceptor is still missing. Therefore, in the present work we produced a uniformly ^13^C-labelled *Cr*phot-LOV1-C57S ($r_{FW}$ ~ 11 Å), aiming to compare the photo-CIDNP effect of FMN and tryptophan under the same conditions. The hyperpolarization effect in combination with isotope labelling paves the way to field-dependent NMR measurements, providing knowledge of the relationship between enhancement factor and magnetic field, which might provide the key data for future theoretical analysis of the exact photo-CIDNP mechanism.

### Comparison of the photo-CIDNP effect between phototropin and aureochrome

**Figure 2:**
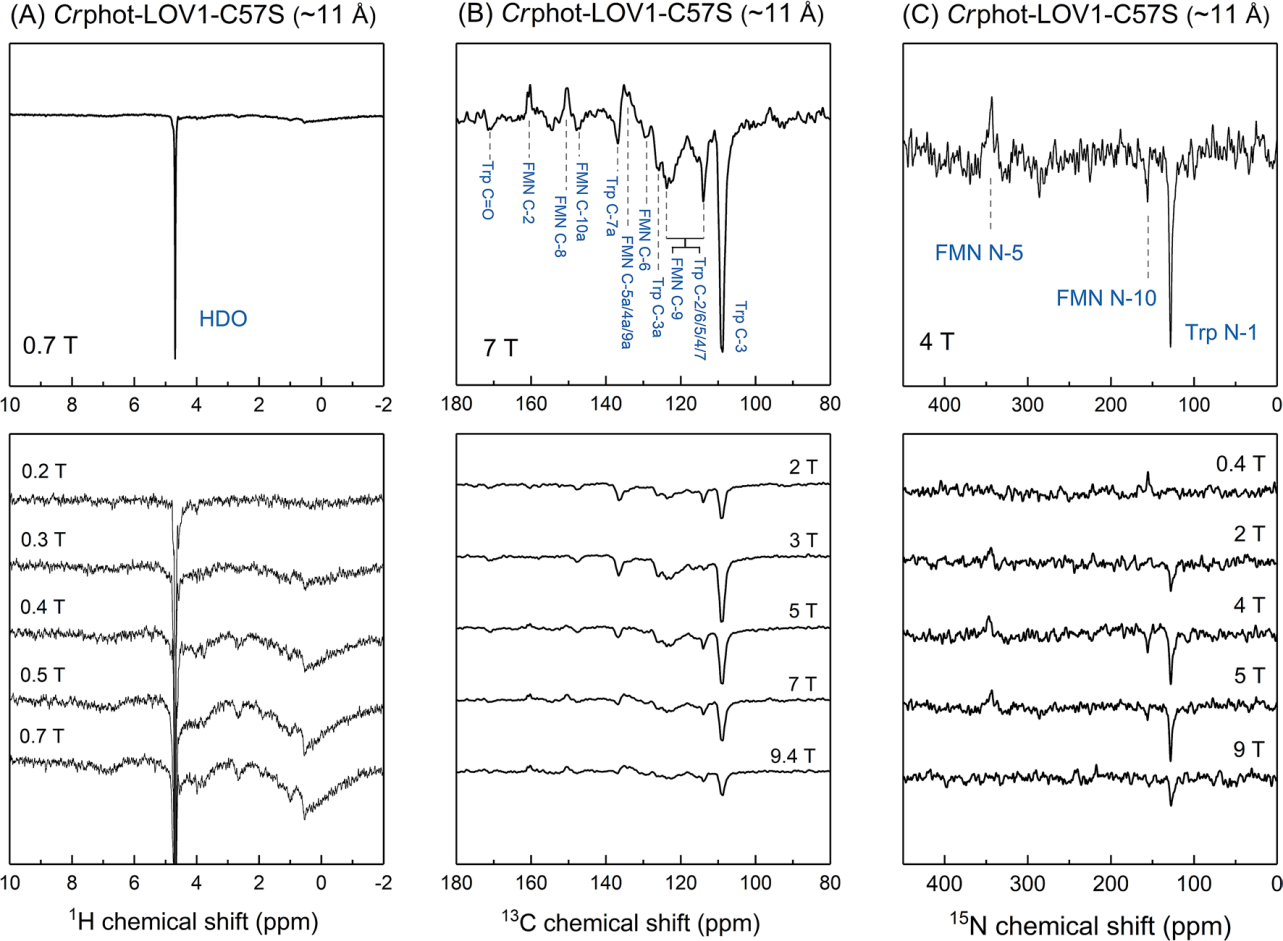
(**A**) ^1^H, (**B**) ^13^C and (**C**) ^15^N photo-CIDNP spectra of *Cr*phot-LOV1-C57S (~ 11 Å) detected at 289 K at different magnetic field strengths by using a 9.4 T liquid-state NMR spectrometer equipped with a field-cycling device. Hereafter, all photo-CIDNP spectra are light-minus-dark NMR spectra. The upper trace includes signal assignments (blue) with ^13^C chemical shifts of hyperpolarized signals mentioned in Supplementary Information Table S2 and ^15^N chemical shifts in Table 2. The lower trace shows stacked spectra of the CIDNP effects of the corresponding nuclei measured at five different magnetic fields.

**Figure 3:**
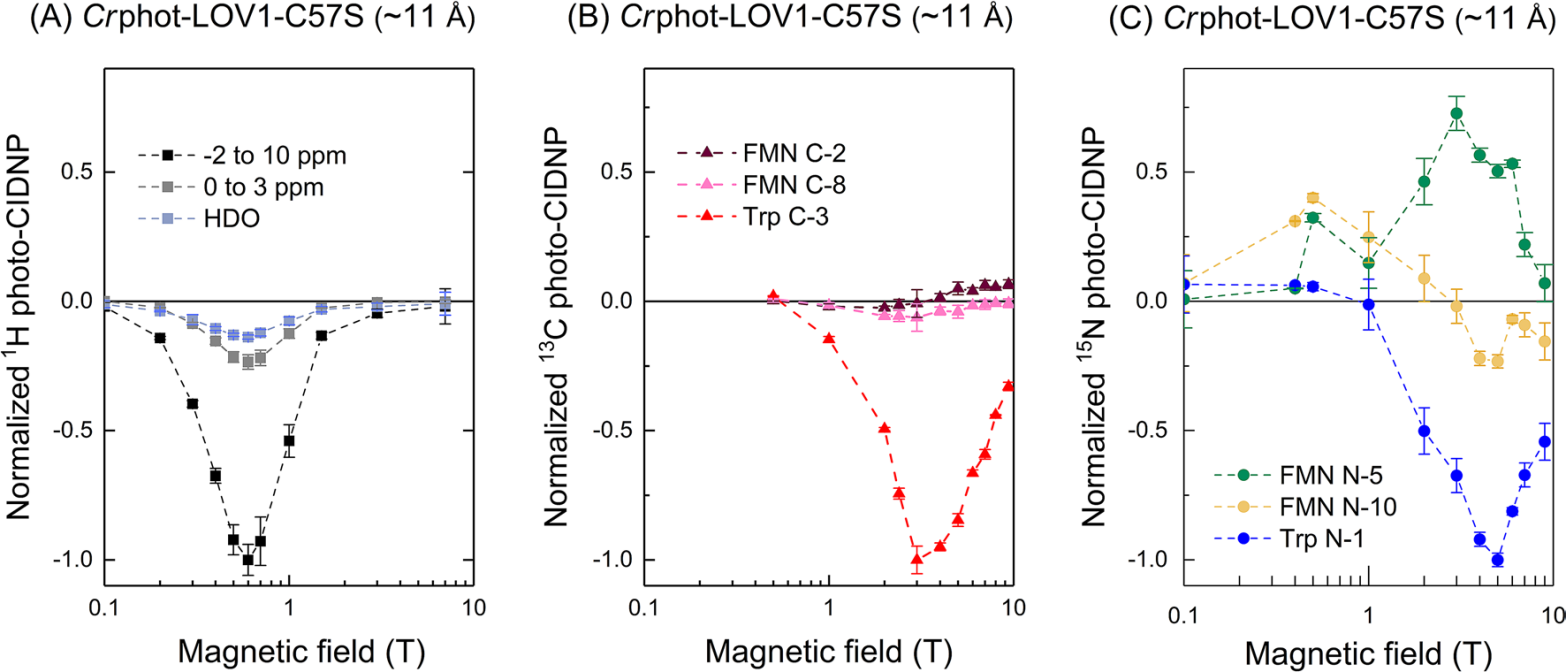
Intensity of selected hyperpolarized signals of *Cr*phot-LOV1-C57S ($r_{FW}$ ~ 11 Å), integrated and plotted against the magnetic field strength for (**A**) ^1^H, (**B**) ^13^C and (**C**) ^15^N. For convenient comparison, the magnitude of the maximum polarization of each nucleus is arbitrarily set to − 1 and the signal enhancement is normalized to this value. The plus and minus signs at the y-axis represent the enhanced absorptive (positive) and emissive polarization (negative) relative to the thermal polarization. Uniformly ^13^C- and uniformly ^15^N-enriched *Cr*phot-LOV1-C57S (~ 11 Å) samples were employed for the ^13^C and ^15^N photo-CIDNP NMR experiments, respectively. Regarding ^1^H photo-CIDNP, the signal of HDO, the aliphatic region (0 to 3 ppm), and the broader region (− 2 to 10 ppm) of the ^1^H photo-CIDNP spectrum were integrated for comparison.

Figure 2A shows the ^1^H photo-CIDNP effect and its field dependence observed in *Cr*phot-LOV1-C57S ($r_{FW}$ ~ 11 Å). The protein produced for the experiment initially contained all the nuclei in their natural abundance. Then the protonated buffer of the sample was exchanged to a deuterated buffer (see “Methods” section). The final protein solution may contain ~ 0.4% residual ^1^H. From this sample, the effect has not been observed directly on the protons of FMN and tryptophan, however, the light-minus-dark spectra show a negative enhancement (emissive signal, i.e., opposite to the thermal polarization) of the HDO signal at 4.7 ppm, which agrees with the previous publication^39^. A closer look at the light-minus-dark spectra shows that the entire range (– 2 to 10 ppm) in the proton NMR spectra exhibits hyperpolarization, in particular the aliphatic region (0 to 2.5 ppm) as well as the HDO signal. Integrating either the HDO signal or the range of 0 to 2.5 ppm or the range of – 2 to 10 ppm, we obtain a field dependence with a maximum at 0.6 T, as shown in Fig. 3A. Since the position of the maximum is the same for all protons, it is plausible that the ^1^H hyperpolarization has originated from the same SCRP and has been distributed over the whole protein as well as to the residual protons in the deuterated solvent. We assume that the spread of hyperpolarization under liquid-state conditions is due to cross relaxation or due to spin diffusion mediated by non-averaged proton-proton dipolar couplings^59,60^.

^13^C photo-CIDNP of a uniformly ^13^C-labelled *Cr*phot-LOV1-C57S ($r_{FW}$ ~ 11 Å) induced at various field strengths ranging from 0.5 to 9.4 T and detected always at 9.4 T is shown in Fig. 2B. The hyperpolarized ^13^C signals are tentatively assigned according to previous studies^39,46,59,61^, and are summarized in Table S2 in Supplementary Information. Figure 3B presents the integrated areas of some selected hyperpolarized carbon signals of FMN and tryptophan against the magnetic field strength. Overall, the carbons on the tryptophan indole ring seem to be stronger polarized than those on the isoalloxazine ring of FMN. The hyperpolarized carbon nuclei of tryptophan show emissive polarization at all fields and maximal polarization at around 3 T in agreement with previous results^39^. The selected ^13^C atoms of the isoalloxazine ring of FMN show different enhancement patterns. As the magnetic field increases, the signal of, e.g., FMN C-8 stays negative, while FMN C-2 changes the sign of polarization from emission to enhanced absorption. Due to the difference of labelling strategies, the comparison of the ^13^C photo-CIDNP spectrum of *Cr*phot-LOV1-C57S ($r_{FW}$ ~ 11 Å) with a previously published ^13^C photo-CIDNP spectrum of *As*phot1-LOV2-C450A ($r_{FW}$ ~ 11 Å)^40,46^ is not straightforward. Despite that, we can compare the signal of Trp C-3 carbon in both spectra. In *As*phot1-LOV2-C450A ($r_{FW}$ ~ 11 Å) where the tryptophan nucleus is at natural abundance, strong hyperpolarization at Trp C-3 occurs. The chemical shift of C-3 of *Cr*phot-LOV1-C57S (109.0 ppm) slightly differs from δ (C-3) = 113.5 ppm for *As*phot1-LOV2-C450A which might result from the different protein environment of the tryptophan residues. In both cases, the sign of the signal of Trp C-3 is always emissive at all fields studied. Nevertheless, the field at which the maximum polarization at Trp C-3 occurs, $B_{max}$ = 3 T for *Cr*phot-LOV1-C57S and $B_{max}$ = 7 T for *As*phot1-LOV2-C450A, is well distinguished. We assume that the difference in the label positions causes the difference in field dependence although details are not yet understood. Furthermore, in a previous work by Kothe et al.^46^ the photo-CIDNP effect has been measured at four different magnetic fields (5.9, 7.1, 9.4, and 11.8 T), limiting comparability of data points and localization of the maximum $B_{max}$, while in the present work, we overcame this problem by using a shuttle system (10 nT < B_o_ < 9.4 T).

Figures 2C and 3C depict the field dependence of the ^15^N photo-CIDNP obtained in uniformly ^15^N-labelled *Cr*phot-LOV1-C57S ($r_{FW}$ ~ 11 Å) at 11 magnetic field strengths ranging from 0.1 to 9.4 T. In Fig. 2C, the three hyperpolarized signals and their field dependencies are shown (for assignment, see Table 2). The Trp N-1 CIDNP is a low absorptive polarization at low fields, turning into strong emissive polarization at about 1 T field and having a maximum at around 5 T. In contrast, the photo-CIDNP of FMN N-5 is always absorptive with a maximum at 3 T. At increasing field, the hyperpolarization of FMN N-10 turns from enhanced absorption to emission at around 3 T, similar to the photo-CIDNP of Trp N-1 and in clear contrast to the photo-CIDNP of FMN N-5. A common feature for these three nuclei is that the high-field maximum occurs at around 4 T.

**Table 2:** Photo-CIDNP experimental results and obtained ^15^N chemical shifts from the eight LOV proteins studied in this work.

Mutants	$r_{FW}$. (Å)	^15^N photo-CIDNP	^1^H photo-CIDNP	^15^N chemical shift (ppm)		
				FMN N-5	FMN-10	Trp N-1
*Cr*Phot-LOV1-C57S	~ 11	+	+	344.1	155.0	127.6
*Pt*Aureo1a-LOV-C287S	~ 11	+	+	n.a.	n.a.	127.8
*Mr*4511-C71S-F130W	~ 6	–	–	n.a.	n.a.	n.a.
*Mr*4511-C71S-Y116W	~ 9	+	+	345.1	156.2	127.7
*Mr*4511-C71S-Q112W	~ 11	+	+	345.3	156.4	127.2
*Mr*4511-C71S-Y129W	~ 11	+	+	345.5	156.2	127.7
*Mr*4511-C71S-K57W	~ 17	+ (new)	+ (new)	345.2	n.a.	n.a.
*Mr*4511-C71S	No Trp	+ (new)	+ (new)	345.3	n.a.	n.a.

*Trp* tryptophan, + tryptophan-derived photo-CIDNP effect, + (*new*) non-tryptophan-derived photo-CIDNP effect, – no photo-CIDNP effect, *n.a.* not available.

**Figure 4:**
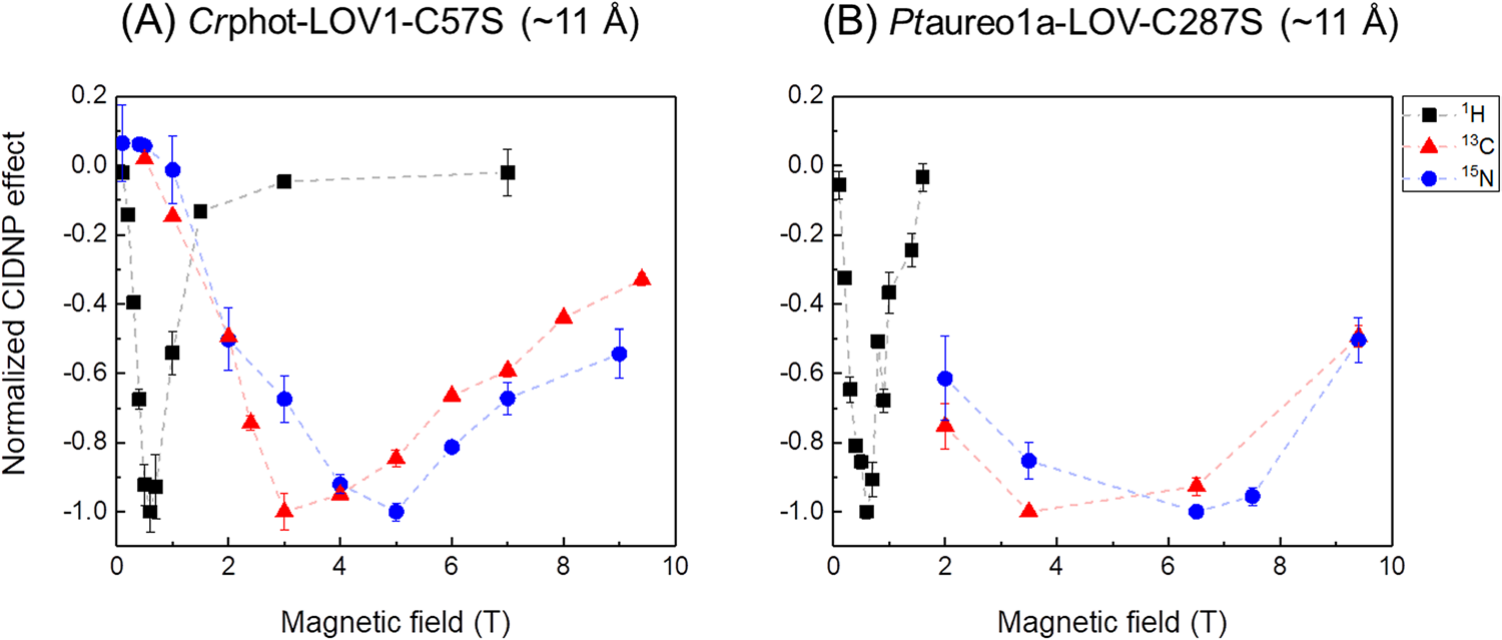
Comparison of the field dependencies of the ^1^H and ^13^C and ^15^N photo-CIDNP effects generated by (**A**) *Cr*phot-LOV1-C57S ($r_{FW}$ ~ 11 Å) and (**B**) *Pt*aureo1a-LOV-C287S ($r_{FW}$ ~ 11 Å). Detailed experimental parameters are provided in the “Methods” section. The hyperpolarized ^1^H signal has been obtained by integrating the spectral region of − 2 to 10 ppm (black square); the ^13^C signal from C-3 of tryptophan (red triangle) and the ^15^N signal from indole of tryptophan (blue circle) are also shown. The dashed lines are added to guide the eye. The maximum emissive polarization of all nuclei is scaled to − 1. The minus sign of the y-axis implies that the hyperpolarization is emissive. The 1D spectra of the photo-CIDNP effect observed in *Pt*aureo1a-LOV-C287S are shown in Supporting Information Figure S1.

For comparison of the photo-CIDNP field dependencies for the three different NMR active nuclear isotopes, we have chosen the most enhanced signal or spectral region for each nucleus, ^1^H, ^13^C and ^15^N: Trp C-3, Trp N-1 and the ^1^H spectrum intensity integrated from – 2 to 10 ppm of *Cr*phot-LOV1-C57S ($r_{FW}$ ~ 11 Å) (Fig. 4A). The field at which the ^1^H, ^13^C and ^15^N photo-CIDNP production reached its maximum follows the relationship: 0.6 T (^1^H) < 3 T (^13^C) < 5 T (^15^N). Under similar conditions, the field maxima for photo-CIDNP enhancement of the corresponding nuclei in *Pt*aureo1a-LOV-C287S ($r_{FW}$ ~ 11 Å) (tryptophan is selectively isotope-labelled) were obtained. The positions of the maxima are in the same order (Fig. 4B); the corresponding 1D spectra are shown in Figure S1. Hence, a high similarity in the field dependence is observed for the two proteins. All three types of nuclei in both samples exhibit emissive NMR signals at the maxima; the positions of maxima depend on the nuclear gyromagnetic ratios (the maximum is positioned at a lower field for a nucleus with a higher gyromagnetic ratio). In both cases, the ^1^H hyperpolarization reaches its emissive maximum at a field around 0.6 T, while the maxima of ^13^C and ^15^N photo-CIDNP appear to be at higher fields. Although the primary sequence of the two proteins does not have a high identity, their tertiary structures (Fig. 1B) and, as shown here, their functional mechanisms are almost identical. Therefore, the production of the photo-CIDNP does not depend crucially on the primary sequence of individual amino acids, whereas the distance between the two redox partners forming the SCRP and their mutual orientation plays of a decisive role.

### Tryptophan and non-tryptophan derived photo-CIDNP effect generated in *Mr*4511 mutants

**Figure 5:**
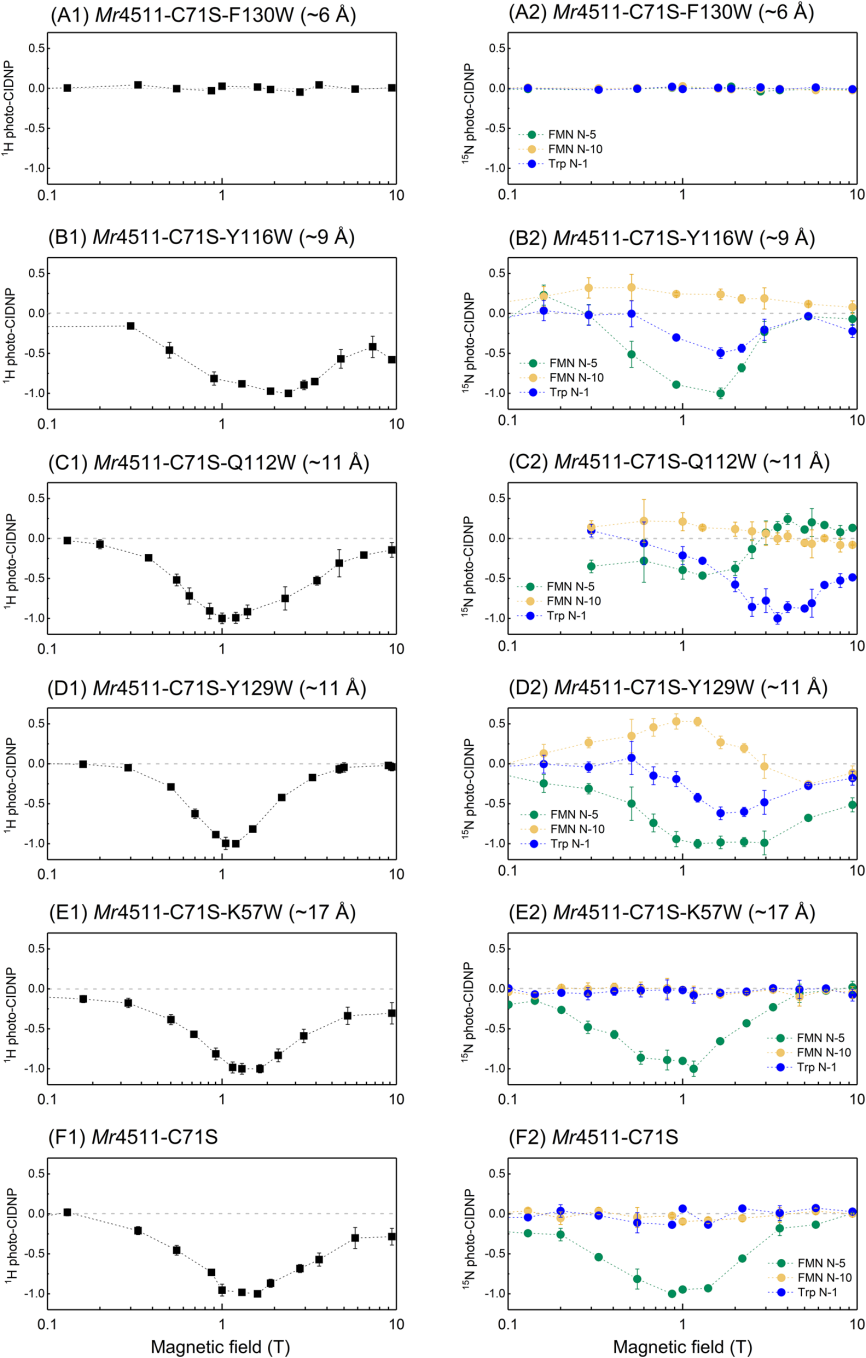
Magnetic-field dependencies of the ^1^H (left column) and ^15^N (right column) photo-CIDNP effect generated in six *Mr*4511-C71S mutants having different $r_{FW}$: (**A**) *Mr*4511-C71S-F130W ($r_{FW}$ ~ 6 Å), (**B**) *Mr*4511-C71S-Y116W ($r_{FW}$ ~ 9 Å), (**C**) *Mr*4511-C71S-Q112W ($r_{FW}$ ~ 11 Å), (**D**) *Mr*4511-C71S-Y129W ($r_{FW}$ ~ 11 Å), (**E**) *Mr*4511-C71S-K57W ($r_{FW}$ ~ 17 Å) and (**F**) *Mr*4511-C71S. Regarding the ^1^H photo-CIDNP, the region of chemical shifts ranging from − 2 to 10 ppm of the spectrum is integrated and plotted against the magnetic field (**A1**–**F1**). The three ^15^N photo-CIDNP hyperpolarized signals correspond to FMN N-5 (green), FMN N-10 (yellow) and Trp N-1 (blue); the signals are plotted against the magnetic field strength (**A2**–**F2**) in the same range and scale as for the ^1^H signal. For straightforward comparison, the signal strength at the maximal hyperpolarization magnitude of each graph is normalized to unity. The positive and negative signs of the y-axis indicate absorptive and emissive hyperpolarization, respectively. Since the mutant F130W shows no detectable photo-CIDNP effect, as presented in the Supplementary Information, Figures S3 and S4, the data points shown in (**A1**) and (**A2**) were measured the over the same spectra regions with the same noise level as in the corresponding spectra for Y129W shown in (**D1**) and (**D2**).

To generate a photo-CIDNP effect in *Mr*4511, the conserved cysteine was first replaced with the inactive serine resulting in the mutant *Mr*4511-C71S. Furthermore, tryptophan was introduced to different locations of the *Mr*4511-C71S protein, generating five additional mutants (Table 1). The magnetic field dependence of the photo-CIDNP effect under liquid-state conditions obtained by ^15^N and ^1^H NMR is summarized in Fig. 5, plotted in the same way as in Fig. 3. The corresponding ^15^N NMR spectra are shown in Figure S2 and the ^15^N chemical shifts are provided in Table 2.

The mutant *Mr*4511-C71S-F130W ($r_{FW}$ ~ 6 Å), which has the shortest distance between the redox partners of the SCRP, shows neither a ^1^H nor a ^15^N photo-CIDNP in the magnetic field range from 0.1 to 9.4 T (Fig. 5A1,A2; Supplementary Information Figure S3 and S4). Time-resolved optical absorption analysis on F130W suggests that the triplet state ^3^FMN is not formed to an observable extent in this protein, most likely, because of the ultra-fast electron transfer between FMN and tryptophan (Aba Losi, personal communication). Accordingly, for the construction of light-induced artificial spin-machines pumping nuclear hyperpolarization, the information of a minimum distance of the redox partners of the SCRP is highly relevant.

Except for *Mr*4511-C71S-F130W ($r_{FW}$ ~ 6 Å), all other *Mr*4511-C71S mutants generated both, the ^15^N and ^1^H photo-CIDNP effect under liquid-state conditions. As shown in Fig. 5B2, *Mr*4511-C71S-Y116W ($r_{FW}$ ~ 9 Å) shows hyperpolarization for the nitrogen on FMN and tryptophan, therefore it is referred to as “tryptophan-derived photo-CIDNP”. This protein shows a maximal ^1^H hyperpolarization at 2.4 T (Fig. 5B1), which is a higher field than observed for any other mutant (see below).

The $r_{FW}$ value of ~ 11 Å in the phototropin and aureochrome LOV domains was known to generate a photo-CIDNP effect^40,41^. Here we compare the two cases when the tryptophan is at the canonical position, *Mr*4511-C71S-Q112W ($r_{FW}$ ~ 11 Å) (Fig. 5C1,C2) and the non-canonical position but with nearly the same $r_{FW}$ distance, *Mr*4511-C71S-Y129W ($r_{FW}$ ~ 11 Å) (Fig. 5D1, D2). Although *Mr*4511-C71S-Q112W and *Mr*4511-C71S-Y129W exhibit a similar field maximum for the ^1^H photo-CIDNP effect, their field-dependent ^15^N photo-CIDNP effects are significantly different. This means that the efficiency of photo-CIDNP formation does not only depend on the spatial distance. Different orientations and different local mobility of the residues might be considered as the origin of this difference. However, the phase of hyperpolarized Trp N-1 signal is allways negative for mutants Y116W, Y129W and Q112W.

The tryptophan residue in *Mr*4511-C71S-K57W ($r_{FW}$ ~ 17 Å) is the most remote electron donor from FMN in the studied set of mutants and, thus, the reaction rate constant of electron transfer in this protein mutant is expected to be the smallest in this series^62^. The protein shows a ^15^N hyperpolarization solely on the FMN N-5, while the FMN N-10 and Trp N-1 signals do not exhibit any enhancement in a wide range of magnetic fields (Fig. 5E). The same photo-CIDNP experiments were also performed on *Mr*4511-C71S in which no tryptophan was present leading to very similar results (Fig. 5F) with somewhat weaker single emissive FMN N5 signal (Figure S2 graphs D and E). This surprising result clearly indicates that the ^15^N photo-CIDNP effect reported here is not derived solely from the involvement of the added tryptophan residue. It is well-known that tyrosine can also act as an electron donor in biological systems^63,64^. According to the amino-acid sequence and our structural model of *Mr*4511, there are four tyrosine residues located in proximity to FMN in the range of 9 to 12 Å. In line with this speculation is the fact that the ^15^N photo-CIDNP effect is not observable for tyrosine, since tyrosine does not contain ^15^N in the side chain. Therefore, the light-driven molecular spin-machines can probably also rely on SCRPs containing a tyrosine radical.

## Discussion

Here, we show that a photo-CIDNP effect originating from the SCRP of FMN and tryptophan can be produced in artificially designed flavoproteins. We employ a systematic mutation strategy to vary reaction and magnetic parameters of the paramagnetic centers generated by light. It appears that the $r_{FW}$ distance of ~ 6 Å between the FMN and tryptophan is too short to provide conditions suitable for photo-CIDNP formation, whereas in the range of ~ 9 Å to ~ 11 Å, the effect has been observed (Fig. 5; Table 2). The data on the field dependence of the photo-CIDNP effect generated by the designed LOV domains show complex dependencies, which are not expected for the liquid-state photo-CIDNP effect. In particular, the differences in field dependence obtained for LOV domains having the same distance of donor and acceptor suggest that anisotropic spin interactions come into play as they are expected for solids. In addition to the field dependence, a distance dependence has been documented. Apparently, further parameters are involved, presumably the relative orientation of donor and acceptor as well as their local dynamics. Both, anisotropy and relaxation effects require further studies. Furthermore, the effect of different label patterns requires a future study.

The presence of solid-state mechanisms in LOV domains in liquids implies that the transient SCRP occurs in a slow-motion regime, during which the anisotropic electron–nuclear interactions are conserved for the build-up of hyperpolarization. In contrast, on NMR time scale, all the anisotropic nuclear interactions, i.e., nuclear dipolar coupling and chemical shift anisotropy as present in solids are averaged out and thus the hyperpolarized signals in the NMR spectra exhibit no obvious anisotropic features. Such phenomenon was previously observed for a photosynthetic RC protein-membrane complex corresponding to ~ 1 MDa, measured by ^13^C liquid-state NMR^28^. For LOV proteins, having the molecular weight of less than 20 kDa, the occurrence of anisotropic mechanisms in liquids likely relies on the formation of dimers^53,57,65^ or higher multimers in solution. The slow tumbling rate may lead to the presence of residual proton-proton couplings which allow for the ^1^H hyperpolarization transfer from the center of the photo-reaction into the environment (Fig. 2A).

So far, flavoproteins and photosynthetic RCs are the only reported electron transfer protein systems that show solid-state photo-CIDNP effect. Even despite the different cofactor arrangements and spin-dynamics, they might share the same mechanisms. Consequently, similar features of CIDNP are expected regarding the sign change of nuclear spin hyperpolarization and the similar field at which the maximum polarization occurs^22,23^. LCs and LACs analysis suggested that a solid-state photo-CIDNP effect is not only field-dependent, but also strongly orientation-dependent because of the anisotropic interactions governing in spin dynamics of the SCRP in solids^36,37^. To the present experiments conducted under liquid-state conditions, the same theory will be applied to understand the sign change that occurred in the LOV proteins as shown in Fig. 3B,C as well as Fig. 5B2–D2.

Summarizing these considerations, we can propose the following interpretation of the experimental findings.

By increasing $r_{FW}$, we decrease two parameters: The SCRP recombination rate and the electron–electron spin–spin coupling, $J_{SCRP}$, within the SCRP. When the $r_{FW}$ distance is too short, photo-CIDNP formation is suppressed, most probably, due to two reasons: The first reason is that $J_{SCRP}$ is too large, introducing an energy gap between the singlet and triplet SCRP spin states. This gap cannot be overcome by the relatively small hyperfine couplings, and singlet–triplet interconversion in the SCRP is thus suppressed. The second reason is that the spin-evolution of the SCRP requires sufficient time for photo-CIDNP formation: fast SCRP recombination interrupts this process and thus no photo-CIDNP is formed.

As $r_{FW}$ increases further, we enter the regime in which the SCRP lifetime is sufficiently long and $J_{SCRP}$ is sizeable, but not too large to suppress singlet–triplet mixing, giving rise to photo-CIDNP formation. In this situation, the TSM scenario comes into play and the maximum position, $B_{max}$, in the photo-CIDNP field dependence is given^37,39^ by the matching condition $\left| {\gamma_{N} } \right|B_{max} \approx J_{SCRP}$, with $\gamma_{N}$ being the nuclear gyromagnetic ratio; the sign of polarization of the three different kinds of nuclei (^1^H, ^13^C and ^15^N) is also consistent with previous theoretical considerations^37,39^. Hence, the $B_{max}$ field is different for different nuclei, which is consistent with the experimental data.

When $r_{FW}$ increases further, $J_{SCRP}$ is decreased and other photo-CIDNP mechanisms^37^ come into play. In this situation, polarization formation is no longer sensitive to the $\gamma_{N}$ value, i.e., to the nuclear spin isotopes, so that different kinds of nuclei exhibit a similar behavior. The sign changes of photo-CIDNP can be rationalized in terms of changing dominance of enhancement mechanisms, as it happens in RCs^37^.

The design strategy also leads to the discovery of a new-type of photo-CIDNP effect generated by *Mr*4511-C71S in which no tryptophan is present. The same effect (Fig. 5E,F) also occurs in *Mr*4511-C71S-K57W ($r_{FW}$ ~ 17 Å). Based on the present results, we are unable to unravel the origin of the new-type photo-CIDNP effect. Recent research on a designed cysteine-lacking *As*phot1-LOV2 domain indicated that, without presence of the tryptophan, the FMN was reduced, however to less extend compared to the case when the tryptophan was present. Kinetic data suggested that one of the tyrosines in the LOV domain acts as counter radical^66^. Therefore, we proposed that tyrosine might act as electron donor in the absence of tryptophan also in our case.

With this, the present work significantly extends the class of light-driven molecular spin machines, which pump nuclear spin-hyperpolarization. The LOV systems are particularly suitable for such biomimetic design, while photosynthetic RCs due to their structural complexity allow for limited manipulations only. The biomimetic design that affects the parameters of the photo-CIDNP effect provides new categories of experiments to analyze the conditions for its occurrence.

## Methods

### Protein preparation

The plasmid (i) encoding the LOV-C287S mutant of aureochrome1a from *P. tricornutum* comprising the flanking Jα and A’α helices (238–378) was received from Peter Kroth (University of Konstanz)^53^. The plasmid (ii) that encodes *Mr*4511 from *M. radiotolerans* (1–164) was generated in our own group by genome cloning^51^. On that basis, we first constructed the cysteine-lacking *Mr*4511-C71S mutant. Subsequently, additional five mutants encoding tryptophan situated at different positions were created, *Mr*4511-C71S-F130W (~ 6 Å), *Mr*4511-C71S-Y116W (~ 9 Å), *Mr*4511-C71S-Q112W (~ 11 Å), *Mr*4511-C71S-Y129W (~ 11 Å) and *Mr*4511-C71S-K57W (~ 17 Å) with primers shown in the Supporting Information Table S3. All genetic manipulations were according to standard protocols. Plasmid (iii) encodes the LOV1-C57S mutant of phototropin from *C. reinhardtii* (16–133) carrying a 15 × His-tag at the *N*-terminus^67^. Further information about all the mutants employed in this work is summarized in Table 1. The protocol of heterologous overexpression and isotope-labelling of all the mutants in *Escherichia coli* has been reported elsewhere^39^. Isotopically enriched material, ^15^NH_4_Cl, [^15^N] indole, [u-^13^C_6_] glucose, and [u-^13^C_8_] indole employed in this research were purchased from Cambridge Isotope Laboratories, Inc. (Andover, MA, USA). Use of ^15^NH_4_Cl or [u-^13^C_6_] glucose as the sole source in the growth medium yields a uniformly ^15^N or ^13^C labelled protein, while supplementation of ^13^C or ^15^N isotope-enriched indole as precursor to the normal medium results in a selective labelling of the tryptophan side chain. The ^15^N and ^13^C labelled proteins were used for corresponding ^15^N and ^13^C NMR measurements. For ^1^H NMR measurement, the employed proteins are in their natural abundance, and they were washed with deuterated phosphate buffer (300 mM NaCl, 50 mM KsPO_4_ in D_2_O, pH 8.0) to a final solution containing approximately 0.4% residual protons. The final concentration of the flavoproteins were controlled at about 16 μM (${\upvarepsilon }_{{450{\text{nm}}}}$ = 12,500 M^−1^ cm^−1^) before photo-CIDNP measurement.

### Photo-CIDNP solution-state NMR

The field-dependent ^15^N-, ^13^C-, and ^1^H photo-CIDNP experiments of the LOV proteins were carried out on an NMR spectrometer operating at a magnetic field of 9.4 T (^1^H frequency of 400 MHz) (Bruker Avance III HD) equipped with a home-built field-cycling device^47^. It transfers the sample to variable magnetic fields within the range 10 nT < B_o_ < 9.4 T at which the sample is illuminated and returns it for the NMR detection at 9.4 T. For the ^13^C and ^15^N photo-CIDNP experiments, pulse-acquire with WALTZ-16 proton decoupling was employed. The pulse sequence of the ^1^H photo-CIDNP experiments starts with a pre-saturation composite pulse sequence^68^ at 9.4 T, followed by the sample shuttle cycle that includes the sample transfer to the chosen magnetic field for illumination by LED (called “light”) or the same cycle without illumination (called “dark”) during the fixed time and the return to 9.4 T, and it ends with the detection sequence. For all photo-CIDNP experiments, the samples were measured in dark and light, respectively, with the same number of scans. The illumination source was a 400-nm 2-W LED (Chanzon, China) and the illumination time was optimized to 0.5 s. A fresh aliquot of the sample stock was taken for a measurement at each magnetic field to compensate the effect of photo-bleaching. The temperature was 289 K for all samples with the exception that *Pt*aureo1a-LOV-C287S was measured at 277 K. The line-broadening for ^15^N and ^13^C NMR spectra were set to 30 Hz and for ^1^H spectra was set to 1 Hz. The ^15^N and ^13^C NMR spectra were phased to the external standard, a mixture of 0.1 M ^15^N labelled urea and 0.1 M ^13^C labelled methanol in DMSO. The chemical shifts of ^15^N NMR spectra were relative to liquid ammonia and referenced externally to urea ^15^N at 76.3 ppm^69^. To present the field-dependence of the photo-CIDNP effect, the selected hyperpolarized signal was integrated and plotted against the field at which the sample was illuminated. For straightforward comparison, the signal strength at the hyperpolarization maximum is set to unity and the other signals are normalized to this value. The positive and negative signs of the y-axis indicate absorptive and emissive hyperpolarization, respectively. The error bars of the ^1^H photo-CIDNP data represent the standard deviation of the mean value obtained from three measurements; the uncertainty of the ^13^C and ^15^N photo-CIDNP data represents the noise level relative to the corresponding hyperpolarized signal area. The spectra shown in Fig. 2 and Supplementary Information Figure S1, S2, and S4 were created with OriginPro Version 2017.

## Supplementary information

Supplementary Information.
