# Supplementary material for: Tailored flavoproteins acting as light-driven spin machines pump nuclear hyperpolarization
Source: Sci Rep. 2020 Oct 29;10:18658. doi: 10.1038/s41598-020-75627-z (PMC7596710; doi:10.1038/s41598-020-75627-z)
Supplement: Supplementary file 1 — Supplementary Information. [file 41598_2020_75627_MOESM1_ESM.pdf]

Supplementary Information

Tailored flavoproteins acting as light-driven spin machines pump nuclear hyperpolarization

Yonghong Ding[§], Alexey S. Kiryutin[†], Ziyue Zhao[§], Qian-Zhao Xu[§&], Kai-Hong Zhao[&], Patrick Kurle[§], Saskia Bannister[‡], Tilman Kottke[‡], Renad Z. Sagdeev[†], Konstantin L. Ivanov[†], Alexandra V. Yurkovskaya[†], Jörg Matysik[§]*

§ Institut für Analytische Chemie, Universität Leipzig, Linnéstr. 3, 04103 Leipzig, Germany

† International Tomography Center, Siberian Branch of Russian Academy of Sciences, Institutskaya, 3a, Novosibirsk, 630090, Russia and Novosibirsk State University, Pirogova 1, Novosibirsk, 630090, Russia

& State Key Laboratory of Agricultural Microbiology, Huazhong Agricultural University, Wuhan 430070, China

‡ Physikalische und Biophysikalische Chemie, Universität Bielefeld, Universitätsstr. 25, 33615 Bielefeld, Germany.

* Correspondence to [joerg.matysik@uni-leipzig.de

This supplementary information contains three tables and four figures.

Table S1. Structural modeling of *Mr*4511 by SWISS-MODEL based on the crystal structure of aureochrome1a- LOV from a eukaryotic photosynthetic stramenopile.

**Model**	This work (Figure 1B)
**Built with**	ProMod3 3.0.0
**Template**	PDB: 3UE6
**Sequence Identity**	48.03%
**Sequence Similarity**	0.42
**Coverage**	0.77
**QSQE**	0.40
**GMQE**	0.62
**QMEAN**	-0.67

Table S2. Chemical shifts assignment of the hyperpolarized ^{13}C signals of FMN and tryptophan obtained from uniformly ^{13}C-labelled *Cr*phot-LOV1-C57S (~11 Å).

**^{13}C chemical shift (ppm)**	**Nuclei**
28.3	Trp C-β
109.0	Trp C-3
114.1	Trp C-7
115.7–124.9	Trp C-2/6/5/4 & FMN C-9
134.5	FMN C-5a/4a/9a
136.6	Trp C-7a
147.7	FMN C-8
150.5	FMN C-10a
160.8	FMN C-2
171.2	Trp C=O

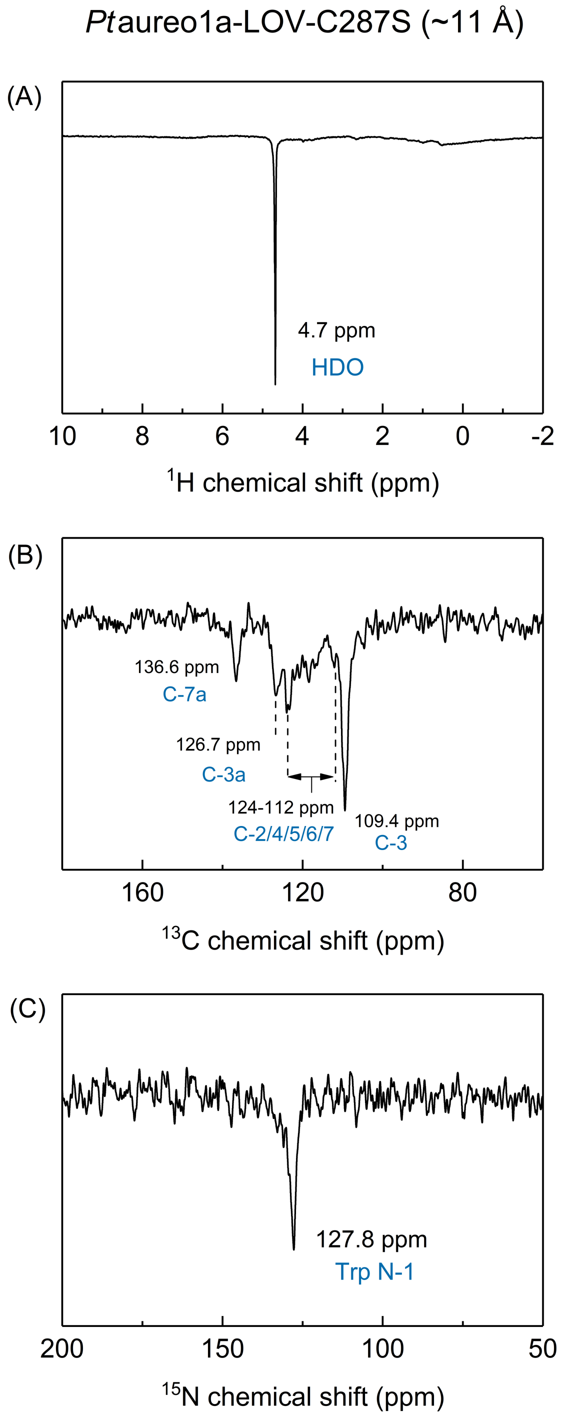

Figure S1. Light-minus-dark difference NMR spectra of the (**A**) ^{1}H, (**B**) ^{13}C, (**C**) ^{15}N photo-CIDNP effect generated by *Pt*aureo1a-LOV-C287S (~11 Å) which is selectively isotope labelled

on the indole moiety of the single tryptophan residue at position 328 with either ^{15}N or with ^{13}C at all positions. The measurement was carried out on a 9.4 T solution NMR equipped with a field-cycling device. The light spectrum of (**A**) was measured at 9.4 T for 128 scans and that of (**B**) at 9.4 T for 384 scans, whereas the spectrum of (**C**) was measured at 0.7 T for 4 scans. The temperature was set to 277 K. The line-broadening for the spectra (**A**) and (**B**) were set to 30 Hz. For spectrum (**C**) it was set to 1 Hz. In (**A**) and (**B**), the hyperpolarized signals are assigned to atoms on tryptophan residue carrying a ^{13}C- and ^{15}N isotope enriched indole moiety. The spectrum (**C**) shows the hyperpolarization on the HDO signal. The same number of scans was recorded under light conditions and in darkness, respectively. The light-minus-dark spectrum is enhanced 10 times for the sake of visibility. The Figure was created with OriginPro Version 2017.

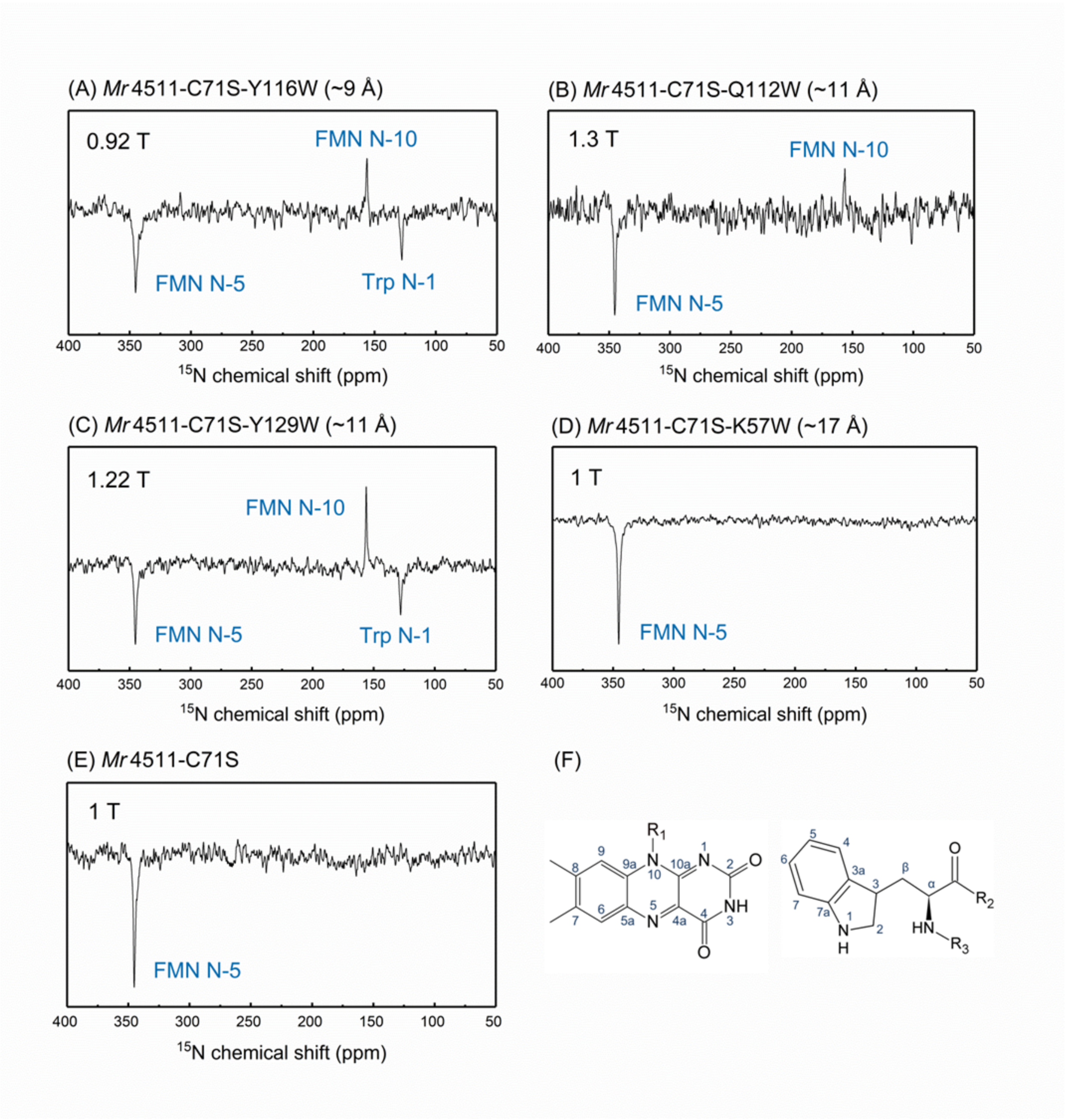

Figure S2. Light-minus-dark ^{15}N NMR spectra of five *Mr*4511 mutants; values in brackets indicate the edge-to-edge distance between the introduced tryptophan residue and the FMN chromophore: (**A**) *Mr*4511-C71S-Y116W (~9 Å) recorded at 0.92 T for 64 scans, (**B**) *Mr*4511-C71S-Q112W (~11 Å) recorded at 1.3 T for 64 scans, (**C**) *Mr*4511-C71S-Y129W (~11 Å) recorded at 1.22 T for 32 scans, (**D**) *Mr*4511-C71S-K57W (~17 Å) recorded at 1 T for 32 scans

and (**E**) *Mr*4511-C71S recorded at 1 T for 16 scans. The temperature was set to 289 K and line-broadening was set to 30 Hz. (**F**) IUPAC numbering of the isoalloxazine ring of FMN and the side chain of tryptophan. The Figure was created with OriginPro Version 2017.

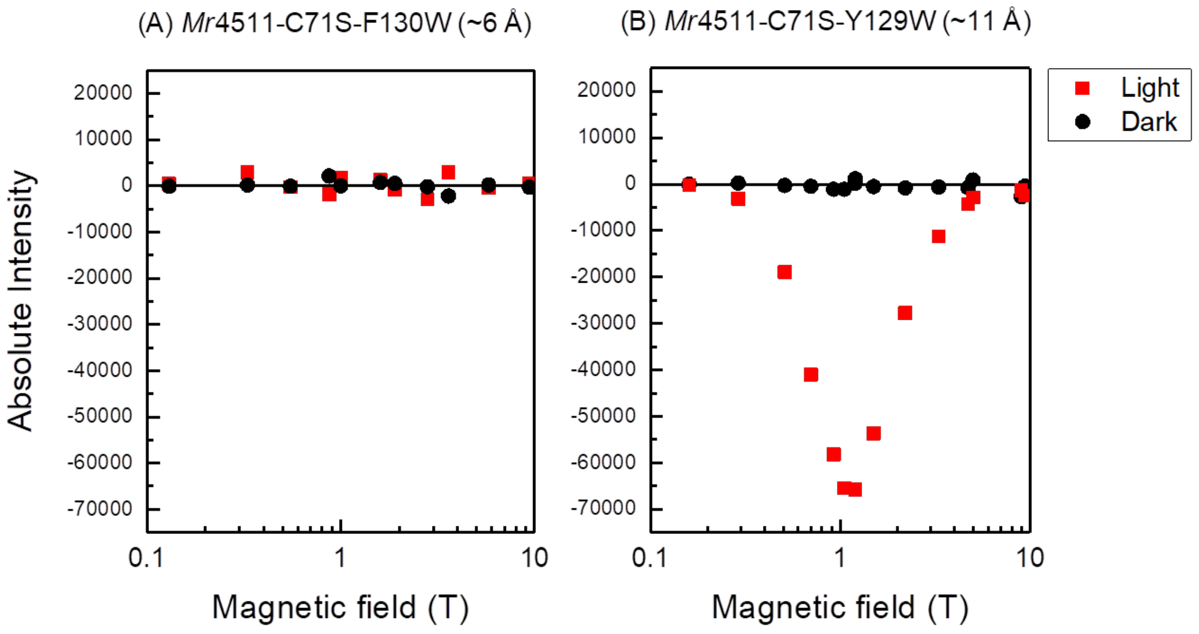

Figure S3. Absolute intensity of the integrated area from 10 to -2 ppm of ^{1}H NMR spectra obtained from(A) *Mr*4511-C71S-F130W (~6 Å) and (B) *Mr*4511-C71S-Y129W (~11 Å) under illumination (red) at various magnetic fields and without illumination (in darkness, black). The comparison of the (A) and (B) indicates that there is no light-induced effect on the ^{1}H spectrum of F130W mutant, which clearly differs from the ^{1}H spectrum of Y129W mutant.

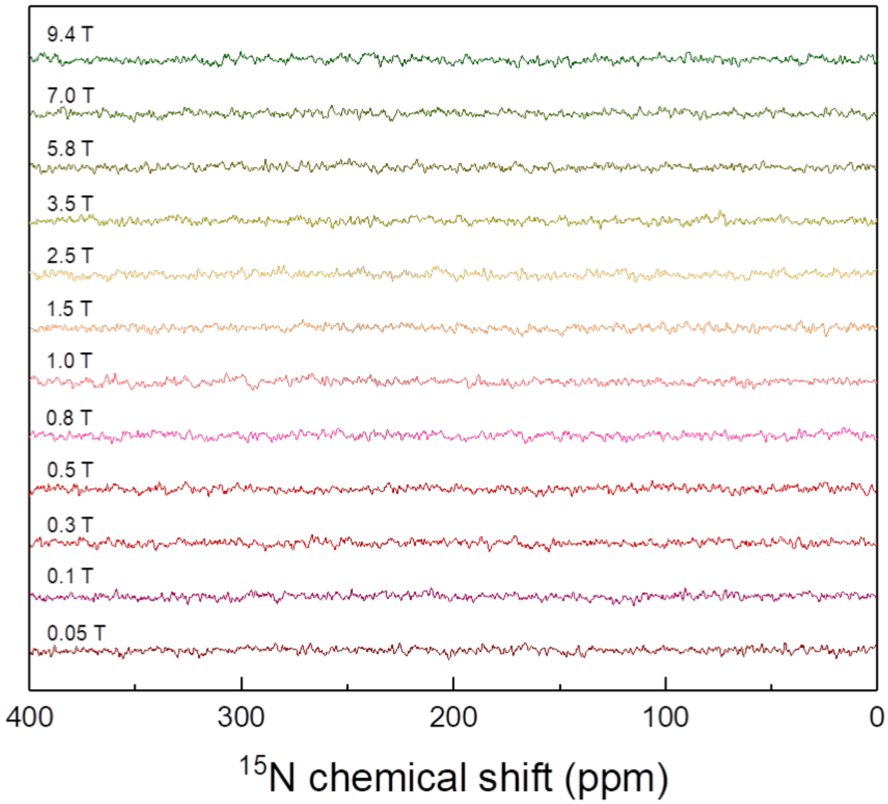

Figure S4. ^{15}N solution NMR spectra of uniformly ^{15}N-labelled *Mr*4511-C71S-F130W (~6 Å) recorded under light. Each spectrum was recorded by illuminating a fresh aliquot of the sample stock at the given magnetic field and measured at 9.4 T. Detailed experimental parameters were provided in the Methods section. The Figure was created with OriginPro Version 2017.

Table S3. Primers for variants of *Mr*4511

Plasmid	Sequence (5' to 3')
Mr4511-C71S	TCCCGCTTCCTGCAGGGG
	GTTGCGGCCGACCACCTCC
Mr4511-C71S-F130W (~6 Å)	GTTCGCCTCGCAGCTCGA
	CAGTAGACCACCCGGCCCG
Mr4511-C71S-Y116W (~9 Å)	TGGGTCGGGCCGGTGC
	GAGGGCGTTCTGGAAGGTCGAG
Mr4511-C71S-Q112W (~11 Å)	GAACGCCCTCTATGTCGG
	CAGAAGGTCGAGCCGTCC
Mr4511-C71S-Y129W (~11 Å)	TGGTTCTTCGCCTCGCAGCT
	GACCACCCGGCCCGCCT
Mr4511-C71S-K57W (~17 Å)	TGGCTGACCGGCTACACCC
	CAGGAACGCGTCGTTCACGAA
